# Generation of KS-133 as a Novel Bicyclic Peptide with a Potent and Selective VIPR2 Antagonist Activity that Counteracts Cognitive Decline in a Mouse Model of Psychiatric Disorders

**DOI:** 10.3389/fphar.2021.751587

**Published:** 2021-11-04

**Authors:** Kotaro Sakamoto, Lu Chen, Tatsunori Miyaoka, Mei Yamada, Teruaki Masutani, Kenji Ishimoto, Nobumasa Hino, Shinsaku Nakagawa, Satoshi Asano, Yukio Ago

**Affiliations:** ^1^ Research and Development Department, Ichimaru Pharcos Company Limited, Gifu, Japan; ^2^ Laboratory of Biopharmaceutics, Graduate School of Pharmaceutical Sciences, Osaka University, Osaka, Japan; ^3^ Laboratory of Innovative Food Science, Graduate School of Pharmaceutical Sciences, Osaka University, Osaka, Japan; ^4^ Global Center for Medical Engineering and Informatics, Osaka University, Osaka, Japan; ^5^ Department of Cellular and Molecular Pharmacology, Graduate School of Biomedical and Health Sciences, Hiroshima University, Hiroshima, Japan

**Keywords:** KS-133, VIPR2/VPAC2, cyclic peptide, bicyclization, antagonist, schizophrenia

## Abstract

Worldwide, more than 20 million people suffer from schizophrenia, but effective and definitive new therapeutic drugs/treatments have not been established. Vasoactive intestinal peptide receptor 2 (VIPR2) might be an attractive drug target for the treatment of schizophrenia because both preclinical and clinical studies have demonstrated a strong link between high expression/overactivation of VIPR2 and schizophrenia. Nevertheless, VIPR2-targeting drugs are not yet available. VIPR2 is a class-B G protein-coupled receptor that possesses high structural homology to its subtypes, vasoactive intestinal peptide receptor 1 (VIPR1) and pituitary adenylate cyclase-activating polypeptide type-1 receptor (PAC1). These biological and structural properties have made it difficult to discover small molecule drugs against VIPR2. In 2018, cyclic peptide VIpep-3, a VIPR2-selective antagonist, was reported. The aim of this study was to generate a VIpep-3 derivative for *in vivo* experiments. After amino acid substitution and structure optimization, we successfully generated KS-133 with 1) a VIPR2-selective and potent antagonistic activity, 2) at least 24 h of stability in plasma, and 3) *in vivo* pharmacological efficacies in a mouse model of psychiatric disorders through early postnatal activation of VIPR2. To the best of our knowledge, this is the first report of a VIPR2-selective antagonistic peptide that counteracts cognitive decline, a central feature of schizophrenia. KS-133 may contribute to studies and development of novel schizophrenia therapeutic drugs that target VIPR2.

## Introduction

Vasoactive intestinal peptide receptor (VIPR) 2, also known as VPAC2, is a class-B G protein-coupled receptor (GPCR) encoded by the *VIPR2* gene. It is expressed in the whole body [e.g., central nervous system (CNS), peripheral nerves, sensory organs, digestive organs, and genital organs] and multifunctions by interacting with two endogenous ligands, vasoactive intestinal peptide (VIP) and pituitary adenylate cyclase-activating polypeptide (PACAP) (Waschek, 2002; Ahrén, 2008; Vaudry et al., 2009; Harmar et al., 2012; Waschek, 2013; Abad and Tan, 2018; Ago et al., 2021). For example, VIPR2 normalizes circadian rhythm (Shen et al., 2000; Harmar et al., 2002; Cutler et al., 2003; Patton et al., 2020), dilates porcine basilar arteries (Grant et al., 2006), stimulates glucose-dependent insulin secretion (Tsutsumi et al., 2002; Giordanetto et al., 2013), and regulates production of proinflammatory cytokines (Tan et al., 2015). More recently, pathological implications of VIPR2 have been shown in psychiatric disorders in humans, such as schizophrenia (Levinson et al., 2011; Vacic et al., 2011; Yuan et al., 2014; Li et al., 2016), autism spectrum disorder (ASD) (Vacic et al., 2011; Firouzabadi et al., 2017), depression (Soria et al., 2010), and attention deficit/hyperactivity disorder (Wilmot et al., 2016). Thus, VIPR2 might be an attractive drug target for the treatment of these diseases. In particular, microduplications at 7q36.3, which contains *VIPR2*, have been strongly associated with schizophrenia with odds-ratios of 14.1 (Vacic et al., 2011) and 6.3 (Levinson et al., 2011). Increased *VIPR2* mRNA expression and cAMP accumulation in response to VIP were observed in cultured lymphocytes of the patients, which demonstrates the functional significance of the microduplications (Vacic et al., 2011). We have previously found that repeated administration of selective VIPR2 agonist Ro 25-1553 to newborn mice reduces synaptic proteins synaptophysin and postsynaptic density protein 95 in the prefrontal cortex and the decline in cognitive functions of mature individuals (Ago et al., 2015). Additionally, we have shown that activation of VIPR2 impairs axon outgrowth and decreases dendritic arborization in mouse cortical neurons (Takeuchi et al., 2020). Tian et al. (2019) developed a conditional human VIPR2 bacterial artificial chromosome transgenic mouse model of *VIPR2* copy number variation (CNV). This mouse model shows cognitive, sensorimotor gating, and social behavioral deficits and decreased complexity of dendritic arborization of striatal spiny projection neurons. In another study, we found that VIPR2-deficient mice exhibit a selective deficit in fear extinction and abnormal dendritic morphology of prefrontal cortex neurons (Ago et al., 2017). These findings suggest that VIPR2 plays an important role in the regulation of dendritic morphology and that the VIPR2 link to mental health disorders may be due in part to overactive VIPR2 signaling at a time when neural circuits involved in cognition and social behavior are being established. Alternatively or additionally, VIPR2 overactivity may disrupt ongoing synaptic plasticity during the processes of learning and memory (Ago et al., 2021). Therefore, a significant potential exists for the development of therapeutics that target this receptor.

Despite these backgrounds, the proof-of-concept of VIPR2 inhibitors has not been examined clinically. A reason might be that VIPR2 belongs to class-B GPCRs and discovery of small molecule drugs against class-B GPCRs is generally difficult (Hollenstein et al., 2014). Another issue is the structural properties of VIPR2. PACAP also binds tightly to its specific receptor PAC1 and high affinity receptors for VIP, namely VIPR1 and VIPR2 (Vaudry et al., 2009; Harmar et al., 2012). VIPR1, VIPR2, and PAC1 have moderate amino acid sequence similarities (about 50%) with each other and highly three-dimensional structural homology (Laburthe et al., 2007). VIP selectively activates VIPR1 and VIPR2, and PACAP activates all three receptors. Therefore, these molecular features have made it difficult to discover VIPR2-selective small molecule drugs (Chu et al., 2010). Under these circumstances, Sakamoto et al. (2018) discovered an artificial 16-mer cyclic peptide VIpep-3, Ac-_c_(CPPYLPRRLC)TLLLRS-OH, which antagonizes human and rodent VIPR2 signaling pathways *in vitro*. VIpep-3 has more than 50-fold receptor selectivity for VIPR2 compared with the two other receptor subtypes. However, this peptide comprises all-natural amino acids and has high susceptibility to degradation by proteases. *In vivo* efficacy of this VIPR2 antagonist also remains to be determined in animal models of psychiatric disorders such as schizophrenia. In this study, we performed amino acid substitutions and structural optimization of VIpep-3 and successfully generated a derivative, KS-133, which possesses the following drug-like properties: 1) selective antagonistic activity against VIPR2 at the nanomolar level, 2) remarkable resistance to protease degradation, and 3) prevention of cognitive decline in a mouse model of psychiatric disorders by early postnatal activation of VIPR2. Here, we show the molecular design, *in vitro* biochemical activities, and *in vivo* pharmacological efficacies of KS-133.

## Materials and Methods

### Synthetic Peptides

All synthetic peptides were synthesized at SCRUM Inc. (Tokyo, Japan) using Fmoc-based solid-phase peptide synthesis, followed by reverse phase-high performance liquid chromatography (RP-HPLC) purification. Peptide purity was ascertained by analytical RP-HPLC and structural assignment was performed by matrix-assisted laser desorption ionization-time of flight mass spectrometry (MALDI-TOF MS). All analytical data of peptides in this report are presented in Supplementary Table S1.

The following briefly describes the synthesis method of KS-133. After synthesis and purification of side chain-protected linear peptide-linked resin, it was dissolved in dichloromethane (DCM) and then mixed with a 2% hydrazine solution for 10 min to deprotect Dde for the side chain of Lys and ODmab for the side chain of Asp. EDC/HOAt was further dissolved in DCM to activate the side chain carboxy group of Asp for amide bond formation with the side chain amide group of Lys. After incubation overnight at room temperature, the resin was washed and then treated with trifluoroacetic acid (TFA) to deprotect the thiol group and excise the monocyclic peptide from the resin. The peptide was purified by RP-HPLC using a SunFire C18 column (Waters Co., Milford, MA, United States). The fraction that contained the product was then collected and lyophilized to produce a side chain-cyclized and -deprotected peptide, Ac-Cys-Pro-Pro-Tyr-Leu-Pro-_c_(Lys-Tyr-Leu-Cys-Asp)-Leu-Ile-NH_2_ (amide bond cyclization with side chains of Lys^7^ and Asp^11^), as a white powder with a mass spectrum of (M–H)^−^ 1561.265 (Calc 1,560.9), purity of 96.14%, and elution time on RP-HPLC (flow rate 1 ml/min) of 11.567 min under linear density gradient elution conditions (A/B = 80/20–10/90 for 20 min using 0.1% TFA in water as eluent A and 0.1% TFA in acetonitrile as eluent B). The peptide was dissolved in 0.1 M NH_4_HCO_3_ (pH 8.0) and reacted for 24–36 h at room temperature to the link side chains of Cys^1^/Cys^10^ by a disulfide bond. The peptide was purified by RP-HPLC using the SunFire C18 column. The fraction with the product was collected and lyophilized to obtain bicyclic peptide KS-133, Ac-_c_[Cys-Pro-Pro-Tyr-Leu-Pro-_c_(Lys-Tyr-Leu-Cys]-Asp)-Leu-Ile-NH_2_ (disulfide bond cyclization with side chains of Cys^1^ and Cys^10^, and amide bond cyclization with side chains of Lys^7^ and Asp^11^) as a white powder whose mass spectrum was (M–H)^−^ 1,558.705 (Calc 1,558.8) with a purity of 100.00% and elution time on RP-HPLC (flow rate 1 ml/min) of 10.617 min under linear density gradient elution conditions (A/B = 80/20–10/90 for 20 min using 0.1% TFA in water as eluent A and 0.1% TFA in acetonitrile as eluent B.

### Calcium Influx and cAMP Assays

Evaluation of antagonist activities of peptides against VIP-VIPR1, VIP-VIPR2, and PACAP-PAC1 signaling pathways and agonistic activity of KS-133 at VIPR2 was carried out by Eurofins DiscoverX (Fremont, CA, United States). Intracellular calcium mobilization was monitored using a calcium-sensitive dye loaded in CHO-K1 cells expressing human VIPR1 (ITEM 86-0030P-2243AN), VIPR2 (ITEM 86-0030P-2244AN), and PAC1 (ITEM 86-0030P-2066AN). The cells were preincubated with antagonistic peptides for 30 min at room temperature in the dark. After addition of the ligand at EC_80_, calcium mobilization was immediately monitored by FLIPR Tetra for 2 min. Cyclic AMP production in CHO-K1 cells expressing human VIPR2 was evaluated using HitHunter^®^ cAMP assays (antagonist mode: ITEM 86-0007P-2362AN; agonist mode: ITEM 86-0007P-2362AG). The cells were preincubated with antagonistic peptides for 30 min at room temperature. After addition of the ligand at EC_80_, cells were further incubated for 30 min at room temperature. Cells were lysed and the cAMP content of the cell lysate was determined by the β-galactosidase-based enzyme fragment complementation assay (Eglen, 2002). IC_50_ values were estimated by the following equation: IC_50_ = 10^[Log(A/B) × (50–D)/(C–D) + Log(B)], where A is the concentration at >50% inhibition, B is the concentration at <50% inhibition, C is the inhibition rate at concertation A, and D is the inhibition rate at concertation B.

### Evaluation of Peptide Stability in Rat Plasma

Each peptide (10 mM, 1 μl) was incubated with rat plasma (20 μl) prepared in-house. Immediately after mixing with plasma and after 24 h of incubation at 37°C, 80% acetonitrile (200 μl) was added to extract VIpep-3 and acetonitrile (200 μl) was added to extract KS-132, KS-133(monocyclic), and KS-133. The mixture was stored for 10 min on ice and then centrifuged at 20,000 × g for 10 min at 4°C. The supernatant was recovered and directly applied to RP-HPLC to determine the remaining amount of unmodified peptides in the sample.

### Animals and Treatments

Experimental procedures that involved animals and their care were conducted in compliance with the *Guide for the Care and Use of Laboratory Animals* (National Research Council, 1996) and ARRIVE guidelines (Kilkenny et al., 2010; McGrath and Lilley, 2015). All animal experiments were approved by the Committee of Research Facilities for Laboratory Animal Science of Hiroshima University (#A20-115) and the Animal Care and Use Committee of the Graduate School of Pharmaceutical Sciences, Osaka University (#30-3-2). Pregnant ICR (CD1) mice at 16 days of gestation and male C57BL/6J mice at 7 weeks of age were purchased from Japan SLC Inc. (Shizuoka, Japan). Mice were housed individually in plastic cages under a standard 12-h light/dark cycle (lights on 08:00 h) at a constant temperature of 22 ± 1°C. The animals had ad libitum access to food and water. Pregnant females were monitored for the parturition date that was considered as postnatal day (P) 0.

For western blot analysis, ICR neonatal mice at P12 (7–10 g body weight) were subcutaneously (s.c.) injected with KS-133 (1 nmol/g) and Ro 25-1553 (0.4 nmol/g, Peptide Institute, Inc. Osaka, Japan), a selective VIPR2 agonist (Gourlet et al., 1997; Harmar et al., 2012), and adult C57BL/6J mice at 8 weeks of age (20–30 g body weight) were intranasally (i.n.) injected with KS-133 (20 nmol/mouse) and BAY 55-9837 (20 µg/mouse, Tocris Bioscience, Bristol, United Kingdom), another selective VIPR2 agonist (Tsutsumi et al., 2002; Tian et al., 2019). The dosages of these drugs were determined in a preliminary experiment using the change in phosphorylation of cAMP-response element-binding protein (CREB) as an index. Ro 25-1553 was dissolved in phosphate-buffered saline (PBS) and BAY 55-9837 was dissolved in distilled water. KS-133 was dissolved in saline with 1% dimethyl sulfoxide (DMSO; #13445-74, Nacalai Tesque, Inc., Kyoto, Japan) for s.c. injection or saline with 5% DMSO and 5 mg/ml poly-L-arginine (#P7762, Sigma-Aldrich, St. Louis, MO, United States) for i.n. injection. The volumes of the drug solutions were 5 ml/kg body weight for s.c. injection and 10 µl for i.n. injection.

For behavioral experiments, all litters were randomly divided into Ro 25-1553- and PBS-treated groups. From P1 to P14, mice were injected s.c. once daily with Ro 25-1553 at a dose of 0.07 nmol/g (Ago et al., 2015). Mice treated with PBS from P1 to P14 were used as the control. KS-133 or the vehicle was simultaneously injected with Ro 25-1553 or PBS. Animals were weaned at P21 and divided by gender at P28. We used male mice exclusively to minimize any potential variability due to sex-specific effects on behavioral performance. All groups were derived from at least four different litters to preclude possible differences in individual maternal behaviors as a mitigating factor in any subsequent long-lasting changes induced in the offspring. Behavioral analyses of mice were carried out at 2−3 months of age. Experimenters were blinded to the treatment while testing.

### Western Blot Analysis

Each mouse was anesthetized with isoflurane and their brain was removed rapidly. The prefrontal cortex was dissected on ice, frozen on dry ice, and stored at −80°C until analysis. Tissue samples were homogenized at 4°C in N-PER™ Neuronal Protein Extraction Reagent (#87792, Thermo Fisher Scientific, MA) with a protease inhibitor cocktail (#25955-11, Nacalai Tesque) and phosphatase inhibitor cocktail (#4906845001, Sigma-Aldrich). The homogenate was incubated for 30 min on ice and then centrifuged at 14,000 × g for 15 min at 4°C and the resulting supernatant was collected. Forty-five (phospho-CREB and CREB) or three (β-actin) micrograms of protein was loaded onto a 4–20% precast gel (#4561096, Bio-Rad Laboratories, Inc., CA), separated by sodium dodecyl sulfate polyacrylamide electrophoresis, and then transferred electrophoretically to a hydrophobic polyvinylidene fluoride membrane. The blotted membranes were blocked with a blocking buffer (5% bovine serum albumin in Tris-buffered saline with 0.1% Tween-20) for 1 h at room temperature and then incubated with primary antibodies phospho-CREB (Ser133) (87G3) rabbit monoclonal antibody (1:500; #9198, Cell Signaling Technology, Danvers, MA), CREB (48H2) rabbit monoclonal antibody (1:1000; #9197, Cell Signaling Technology) at 4°C overnight, or β-actin mouse monoclonal antibody (1:5000; #A2228, Sigma-Aldrich) for 1 h at room temperature. The membranes were then incubated with horseradish peroxidase-conjugated anti-rabbit IgG (1:3000; #7074, Cell Signaling Technology) or anti-mouse IgG (1:5000; #ab6789, Abcam, Cambridge, MA) for 1 h at room temperature. The primary and secondary antibodies were diluted with Can Get Signal™ (#NKB-101T; TOYOBO Co., Ltd., Osaka, Japan) Solution 1 and 2, respectively. Immune complexes were visualized using ECL2 Western Blotting Detection Reagents (PerkinElmer, Inc., Waltham, MA). Densitometric analysis was carried out using the CS analyzer 4 software package (ATTO Co., Kyoto, Japan). Expression levels of phospho-CREB were normalized to total CREB and β-actin, and expression levels of CREB were normalized to β-actin. Data are presented as the fold change relative to the PBS/vehicle-treated control group.

### Novel Object Recognition Test

The novel object recognition test was carried out in accordance with previous reports (Takuma et al., 2014; Hara et al., 2016). In brief, after habituation to the experimental box under dim light conditions (30 lx) for 3 consecutive days, the test mouse was allowed to freely explore two novel objects (A and B) placed in the box for 10 min. Twenty-four hours after the training session, the retention session was conducted. In the retention session, object B was replaced with novel object C and the mouse was allowed to move freely for 5 min in the same box. The exploration time for each object in the retention session was measured with a stopwatch. The discrimination index (%) was the difference between the exploration time for the novel object plus that for the familiar object divided by the total exploration time. This index was used to calculate values for recognition memory. This test was conducted between 9:00–15:00.

### Histology and Dendritic Analyses

Dendritic morphological analysis was performed on mice after the novel object recognition test and samples were collected within 10 min of dissection. Golgi-Cox impregnation was performed using an FD Rapid GolgiStain™ Kit (#PK401, FD Neurotechnologies Inc., Columbia, MD) as described previously (Takuma et al., 2014; Hara et al., 2016; Ago et al., 2017). Briefly, mice were deeply anesthetized with isoflurane, and their brains were removed, rinsed with Milli-Q water, and immersed in impregnation solution composed of potassium dichromate, mercuric chloride and potassium chromate. The brains were stored at room temperature for 2 weeks and then transferred and stored in a cryoprotectant solution for 72 h in the dark. The impregnated brains were embedded in 3.5% agarose gel and cut with a vibratome (VT1000S; Leica Microsystems) at room temperature. Coronal sections of 100 μm in thickness were mounted on Gelatin-Coated Microscope Slides (#PO101, FD Neurotechnologies Inc.) and dried in air at room temperature in the dark for 24 h. After drying, the sections were rinsed with Milli-Q water, reacted in the working solution, and dehydrated with a 50, 75, 95, and 100% graded ethanol series. Finally, the sections were dewaxed in xylene and coverslipped using Mount Quick (Daido Sangyo, Saitama, Japan). Digitized images from the prefrontal cortex (+2.245 through +1.345 mm with respect to the bregma) (Dong, 2008) were obtained under an upright light microscope with a cooled CCD digital camera system (Axio Imager.M2/AxioCam MRc5; Carl Zeiss, Jena, Germany). A 20× lens was used to measure dendrites. Only fully impregnated neurons that displayed dendritic trees without obvious truncation and that were isolated from neighboring impregnated neurons were retained for analysis. Forty pyramidal neurons with the soma in layers II/III were selected in the prefrontal cortex from five mice per group. Morphologies of apical and basal dendrites were quantified in three dimensions using the Neurolucida neuron tracing system (MBF Bioscience, Williston, VT) with the experimenter blinded to the treatment. The total length and branch number of apical and basal dendrites were compared among treatments. To assess differences in the amount and location of dendritic material, Sholl analysis was performed using a NeuroExplorer (MBF Bioscience).

### Data Analysis

All data are expressed as the mean ± standard error of the mean (SEM). Data from western blots were analyzed using one-way analysis of variance (ANOVA), followed by the Tukey–Kramer post-hoc test. For behaviors, the total number of dendrites, and dendritic length, data were analyzed using two-way ANOVA, followed by the Tukey–Kramer test. For Sholl analysis, data were analyzed using two-way ANOVA with drug treatment as the intersubject factor and repeated measures with distance from the soma as the intrasubject factor. Statistical analyses were conducted using the software package StatView^®^ 5.0 for Windows (SAS Institute, Cary, NC). A value of *p* < 0.05 was considered statistically significant.

## Results

### Molecular Design of VIpep-3 Derivatives

**FIGURE 1:**
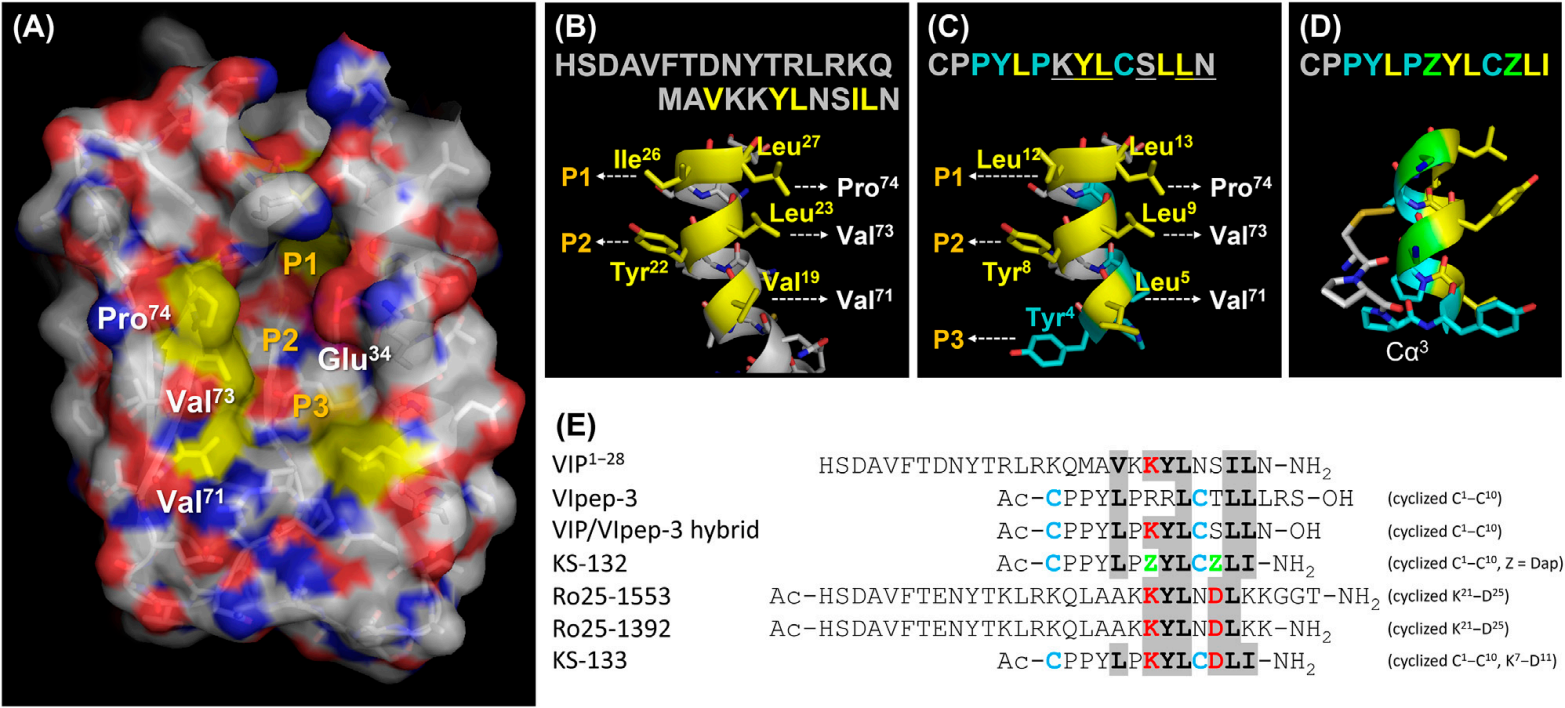
Peptide molecular design strategy. **(A)** Extracellular domain of VIPR2^26−114^ (PDB ID: 2X57). **(B)** C-terminal structure of VIP^1−28^ (PDB ID: 2RRI). **(C)** Structural model of the VIP/VIpep-3 hybrid peptide. **(D)** Proposed mechanism of S-S bond formation on the VIpep-3 derivative. **(E)** Peptide design concept with reference to VIP and analogues. This figure was prepared using PyMOL software.

For the molecular design of VIpep-3 derivatives, it is favorable to have structural information of VIpep-3, Ac-_c_(CPPYLPRRLC)TLLLRS-OH. However, unfortunately, the crystal structure of the VIpep-3/VIPR2 complex has not been obtained to date. Alternatively, three structural aspects, the extracellular domain of VIPR2^26−114^ (PDB ID: 2X57), VIP^1−28^ (HSDAVFTDNYTRLRKQMAVKKYLNSILN-NH_2_) (PDB ID: 2RRI) (Umetsu et al., 2011), and a structural model of the VIP/VIPR1 complex (Couvineau et al., 2012) have been reported. Using this information, we first predicted the interaction mechanism between VIP and VIPR2. As shown in Figure 1A, a deep and wide groove that comprised three pockets (P1–P3) was observed on VIPR2. When we superimposed the structure of VIP onto the groove, Val^19^, Leu^23^, and Leu^27^ of VIP were likely to form hydrophobic interactions with Val^71^, Val^73^, and Pro^74^ of VIPR2 (Figure 1B). Additionally, Ile^26^ and Tyr^22^ of VIP were likely to interact with pocket-1 and pocket-2 of VIPR2, respectively (Figure 1B).

Next, we tried to use this predicted binding mode of VIP for the molecular design of VIpep-3 derivatives. We found that the sequence order pattern of aliphatic amino acids of VIpep-3 (Leu^5^, Leu^9^, Leu^12^, and Leu^13^) was similar to that of VIP (Val^19^, Leu^23^, Ile^26^, and Leu^27^) (Figure 1E). Therefore, we hypothesized that VIpep-3 had a similar secondary structure as VIP and then designed a VIP/VIpep-3 hybrid sequence, CPPYLPKYLCSLLN (underlined amino acids are from VIP and others are from VIpep-3). When we superimposed the structure onto the groove of VIPR2, Tyr^4^ was likely to interact with pocket-3 (Figure 1C) in addition to the predicted binding mode of VIP/VIPR2. A disulfide bond between Cys^1^ and Cys^10^ could be formed by 180° reverse orientation of the backbone at the point of Cα^3^ (Figure 1D). This was supported by the fact that the reported VIpep-3 family has an extremely conserved Pro^6^ (Sakamoto et al., 2018), which is a 5-membered ring heterocycle amino acid without a hydrogen bond donor Nα and makes it possible to flip the main chain orientation by breaking the helix structure.

The amino acid sequence of VIpep-3 is highly conserved in its family except for Arg^7^, Arg^8^, Thr^11^, Leu^14^, Arg^15^, and Ser^16^ (Sakamoto et al., 2018). On the basis of the predicted binding mode, it appeared to be better to substitute Arg^8^ with Tyr. Because the three C-terminal residues Leu^14^, Arg^15^ and Ser^16^ were unlikely to be deeply involved in VIPR2-binding (Sakamoto et al., 2018), they were deleted. Consequently, the remaining amino acid-substitutable residues were Arg^7^ and Thr^11^.

**FIGURE 2:**
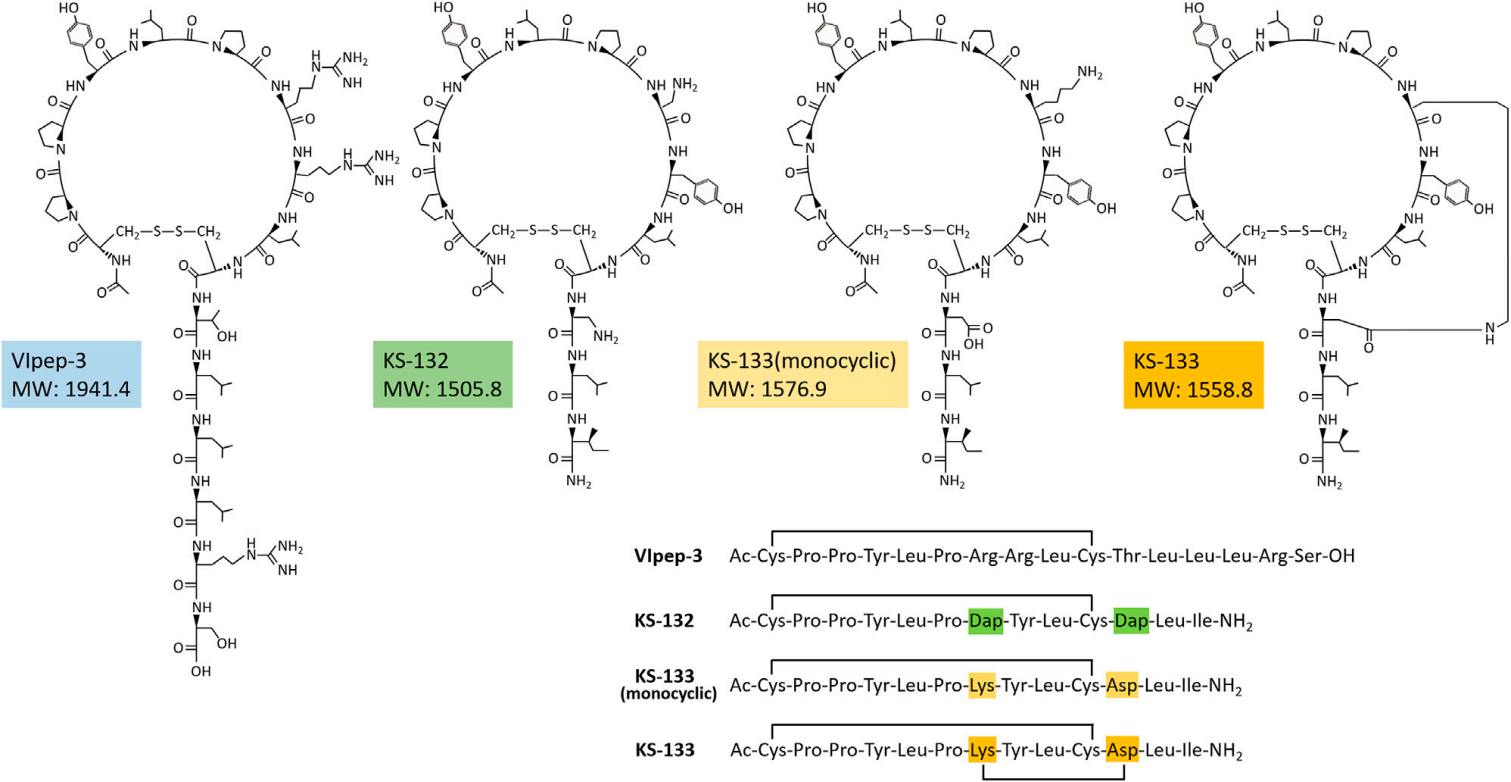
Chemical structures of peptides.

A strategy for VIpep-3 structure optimization is introduction of positively charged amino acid L-2,3-diaminopropionic acid (Dap) to the 7^th^ and 11^th^ positions to interact with negatively charged Glu^34^ of VIPR2. KS-132, Ac-_c_(Cys-Pro-Pro-Tyr-Leu-Pro-Dap-Tyr-Leu-Cys)-Dap-Leu-Ile-NH_2_ (disulfide bond cyclization with side chains of Cys^1^ and Cys^10^), was designed and synthesized (Figures 1E, 2).

Another strategy is bicyclization. In accordance with previous studies on VIP structure optimization, cyclization by bridging between the 21^st^ and 25^th^ positions was effective (Gourlet et al., 1997; Yung et al., 2003; Onoue et al., 2008; Giordanetto et al., 2013). For example, VIP analogues Ro 25-1553 (Gourlet et al., 1997) and Ro 25-1392 have been reported as VIPR2-selective agonists that have a cyclic structure by bridging between Lys^21^ and Asp^25^ (Figure 1E). Therefore, we chose bicyclization as another strategy. KS-133, Ac-_c_[Cys-Pro-Pro-Tyr-Leu-Pro-_c_(Lys-Tyr-Leu-Cys]-Asp)-Leu-Ile-NH_2_ (disulfide bond cyclization with side chains of Cys^1^ and Cys^10^, and amide bond cyclization with side chains of Lys^7^ and Asp^11^), was designed and synthesized (Figures 1E, 2). This bicyclization structure should stabilize active conformation of peptide.

### *In Vitro* Antagonistic Activities of VIpep-3 Derivatives Towards PACAP Receptors

**FIGURE 3:**
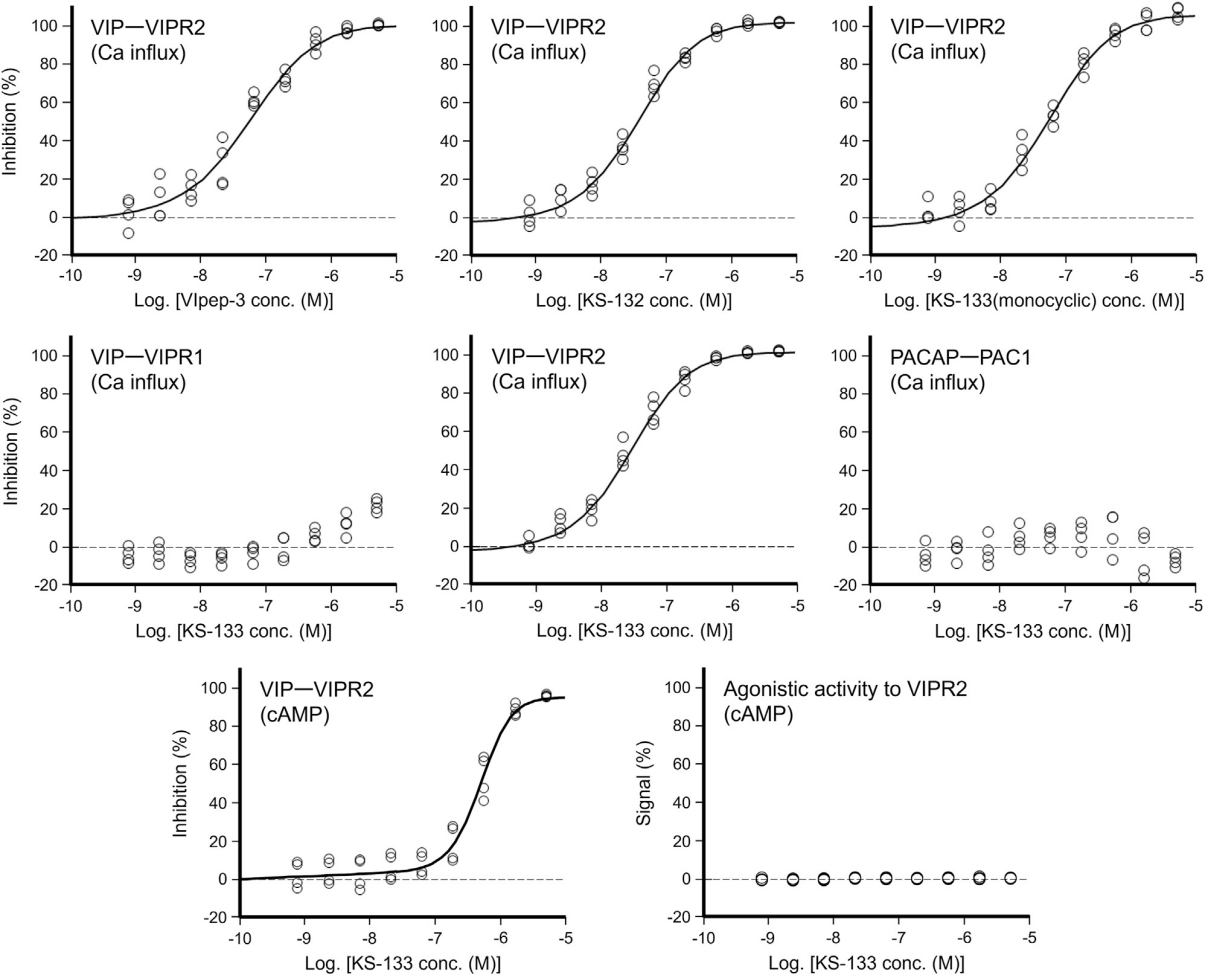
Antagonistic/agonistic activities of peptides in the calcium influx and cAMP assays. For evaluation of the antagonistic activity, the cells were preincubated with antagonistic peptides for 30 min. In the calcium influx assay, after addition of ligand at EC_80_, calcium mobilization was monitored immediately (*see* details in the Methods section) (*n* = 4 wells for each treatment). In the cAMP assay, after a 30 min incubation with the ligand at EC_80_, cAMP production in cells was measured (*see* details in the Methods section) (*n* = 4 wells for each treatment).

First, the antagonistic activities of parental VIpep-3, KS-132, KS-133 (monocyclic) (no bridging between Lys^7^ and Asp^11^), and KS-133 against human VIPR1, VIPR2, and PAC1 were evaluated by a calcium influx assay. After antagonistic peptide treatment for 30 min, ligand at a concentration of EC_80_ was added to the cells and then calcium signaling was measured. All peptides antagonized the VIP-VIPR2 signaling pathway in a peptide concentration-dependent manner (Figure 3) but did not antagonize VIP-VIPR1 or PACAP-PAC1 signaling pathways up to 5 μM (Table 1). KS-132 and KS-133 had IC_50_ values of 33.4 and 24.8 nM, respectively, which were stronger antagonistic activities than parental VIpep-3 (40.6 nM) (Table 1). This result suggested that both of the abovementioned two molecular design strategies were successful. Additionally, this result indicated that these derivatives had inherited the molecular properties required for VIPR2-selective binding activity from parental VIpep-3.

**TABLE 1:** Antagonistic/agonistic activities of peptides in the calcium influx and cAMP assays.

	Ca influx assay [IC_50_ (nM)]			cAMP assay [IC_50_ (nM)]	cAMP assay (nM)
	VIP (25 nM)	VIP (150 nM)	PACAP (35 nM)	VIP (0.5 nM)	Agonistic activity
	VIPR1	VIPR2	PAC1	VIPR2	VIPR2
VIpep-3	>5,000	40.6 ± 4.0	>5,000	N.T.	N.T.
KS-132	>5,000	33.4 ± 2.8	>5,000	N.T.	N.T.
KS-133(monocyclic)	>5,000	56.0 ± 3.9	>5,000	N.T.	N.T.
KS-133	>5,000	24.8 ± 3.2	>5,000	500 ± 79	>5,000

Peptides were tested in the presence of EC_80_ ligand (*n* = 4 wells, ± S.E.M).

N.T. means not tested.

Next, antagonistic and agonistic activities of KS-133 at VIPR2 were evaluated by a cAMP assay. As shown in Figure 3, KS-133 antagonized VIP-VIPR2 signaling pathway in a peptide concentration-dependent manner. The IC_50_ value was determined as 500 nM (Table 1). On the other hand, KS-133 did not exhibit VIPR2 agonist activity, even at high concentration of 5 μM (Figure 3 and Table 1).

### Resistance to Protease Degradation of VIpep-3 Derivatives

The stability of peptides was evaluated in rat plasma. A peptide was incubated in rat plasma at 37°C for 0 and 24 h and the remaining amount of peptide was determined by RP-HPLC. By comparison with the peak area of the peptide, the remaining amount of the peptide after plasma incubation was estimated. As shown in Table 2, monocyclic peptides VIpep-3 and KS-132 were significantly degraded within 24 h. Conversely, bicyclic peptide KS-133 was highly stable for at least up to 24 h. Interestingly, KS-133(monocyclic) was also highly stable. KS-133(monocyclic) may form a pseudo bicyclic structure by ionic bond interaction between side chains of Lys and Asp.

**TABLE 2:** Stabilities of peptides in rat plasma.

Peptide name	Input (NT)	Immediately after mixing	Incubation time 24 h
VIpep-3	1299273	900813	95354
		100%	10.6%
KS-132	1460241	1206247	696652
		100%	57.8%
KS-133(monocyclic)	768775	744262	724176
		100%	97.3%
KS-133	161695	165254	163537
		100%	99.0%

Peak area values obtained by RP-HPLC and the residual rate (%) are listed.

NT indicates no plasma treatment.

### *In Vivo* VIPR2 Antagonistic Activity of KS-133

**FIGURE 4:**
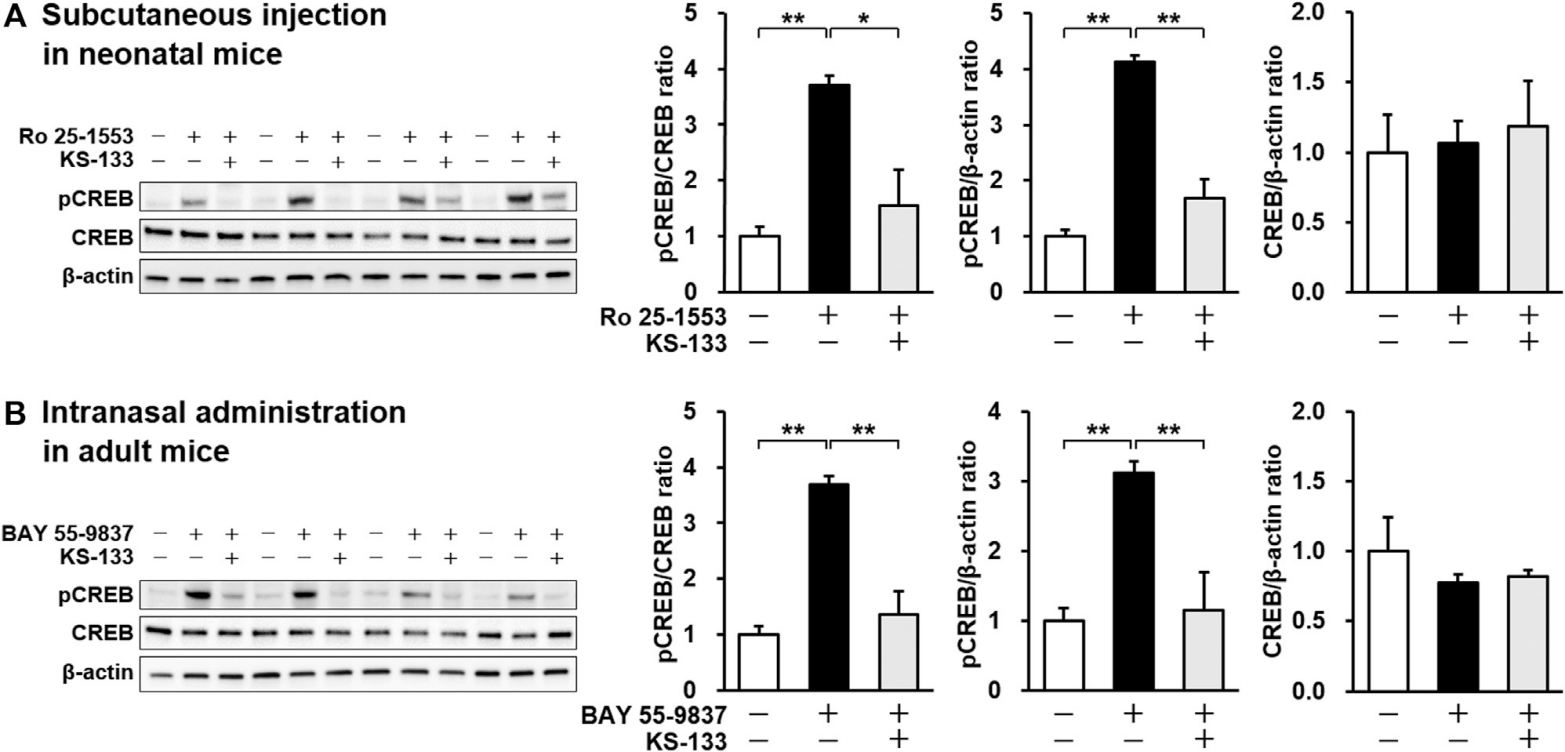
Effects of subcutaneous **(A)** and intranasal **(B)** administration of KS-133 on VIPR2 activation-induced phosphorylation of CREB in the prefrontal cortex of mice. Phospho-CREB (pCREB), total CREB, and β-actin levels were analyzed by western blotting. **(A)** Mice at P12 were s.c. injected with Ro 25-1553 (0.4 nmol/g) or PBS. KS-133 (1 nmol/g) or the vehicle were injected simultaneously into mice. One hour after the injection, the prefrontal cortex of mice was isolated and pCREB was examined. **(B)** Mice at 8 weeks of age were i.n. injected with BAY 55-9837 (20 µg/mouse) or distilled water. KS-133 (20 nmol/mouse) or the vehicle were injected simultaneously into mice. Forty-five minutes after the injection, the prefrontal cortex of mice was isolated and pCREB was examined. Expression levels of pCREB were normalized to total CREB and β-actin. Results are expressed as the mean ± S.E.M. of four mice per group. *p < 0.05, **p < 0.01.

KS-133, which had the strongest antagonistic activity *in vitro* and good resistance to protease degradation, was selected for *in vivo* evaluation. To determine whether KS-133 blocked VIPR2-mediated signaling in the brain, we first examined the effects of systemic administration of KS-133 on phosphorylation of CREB, a biomarker downstream of VIPR2, in neonatal and adult mice (Figure 4). Subcutaneous administration of selective VIPR2 agonist Ro 25-1553 (0.4 nmol/g) to ICR mice at P12, when VIPR2 is highly expressed in mouse brain (Waschek et al., 1996), significantly enhanced phosphorylation of CREB in the prefrontal cortex (Figure 4A). Simultaneous injection of KS-133 (1 nmol/g) suppressed the Ro 25-1553-induced increase in CREB phosphorylation. We also evaluated the effects of KS-133 in adult mice (Figure 4B). Intranasal administration of selective VIPR2 agonist BAY 55-9837 (20 µg/mouse) significantly increased phosphorylated CREB in the prefrontal cortex, which was blocked by coadministration of KS-133 (20 nmol/mouse) with BAY 55-9837.

**FIGURE 5:**
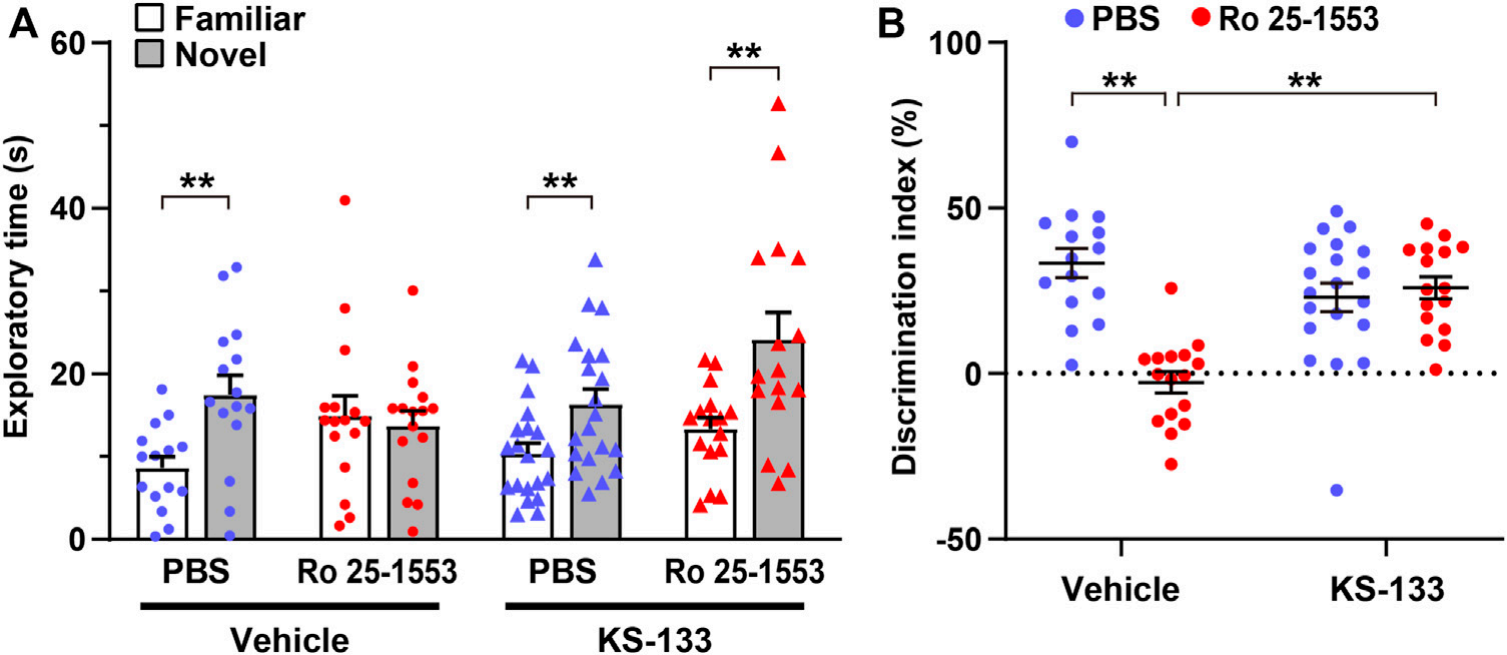
Effects of early postnatal treatment with Ro 25-1553 and KS-133 on recognition memory in the novel object recognition test. Mice were injected s.c. once daily with Ro 25-1553 (0.07 nmol/g) or PBS from P1 to P14. KS-133 or the vehicle was simultaneously injected s.c. with Ro 25-1553 or PBS. Then, the novel object recognition test was conducted in adulthood. **(A)** Time spent exploring each object in the test session. **(B)** Discrimination index (%) was calculated as the difference between novel and familiar object exploration times divided by the total time spent exploring the two objects. Results are expressed as the mean ± S.E.M. of 15–20 mice per group. **p < 0.01.

Next, we investigated the pharmacological efficacy of KS-133 in a mouse model of psychiatric disorders on the basis of early postnatal activation of VIPR2 (Ago et al., 2015) (Figure 5). Repeated administration of Ro 25-1553 (0.07 nmol/g, s.c., once daily) during P1–14 significantly decreased the difference in time spent exploring each object, whereas PBS-treated control mice explored the novel object by a significant preference in the novel object recognition test (Figure 5A). Additionally, the discrimination index was significantly lower in mice treated with Ro 25-1553 than in control mice (Figure 5B). Chronic treatment with KS-133 (1 nmol/g) attenuated the decreases in the difference of time spent exploring each object and cognitive dysfunction. Two-way ANOVA of the discrimination index revealed significant main effects of Ro 25-1553 treatment (*F*_1,63_ = 17.378, *p* < 0.0001) and KS-133 treatment (*F*_1,63_ = 5.261, *p* < 0.05), and there was a significant interaction between Ro 25-1553 and KS-133 treatments (*F*_1,63_ = 23.988, *p* < 0.0001).

**FIGURE 6:**
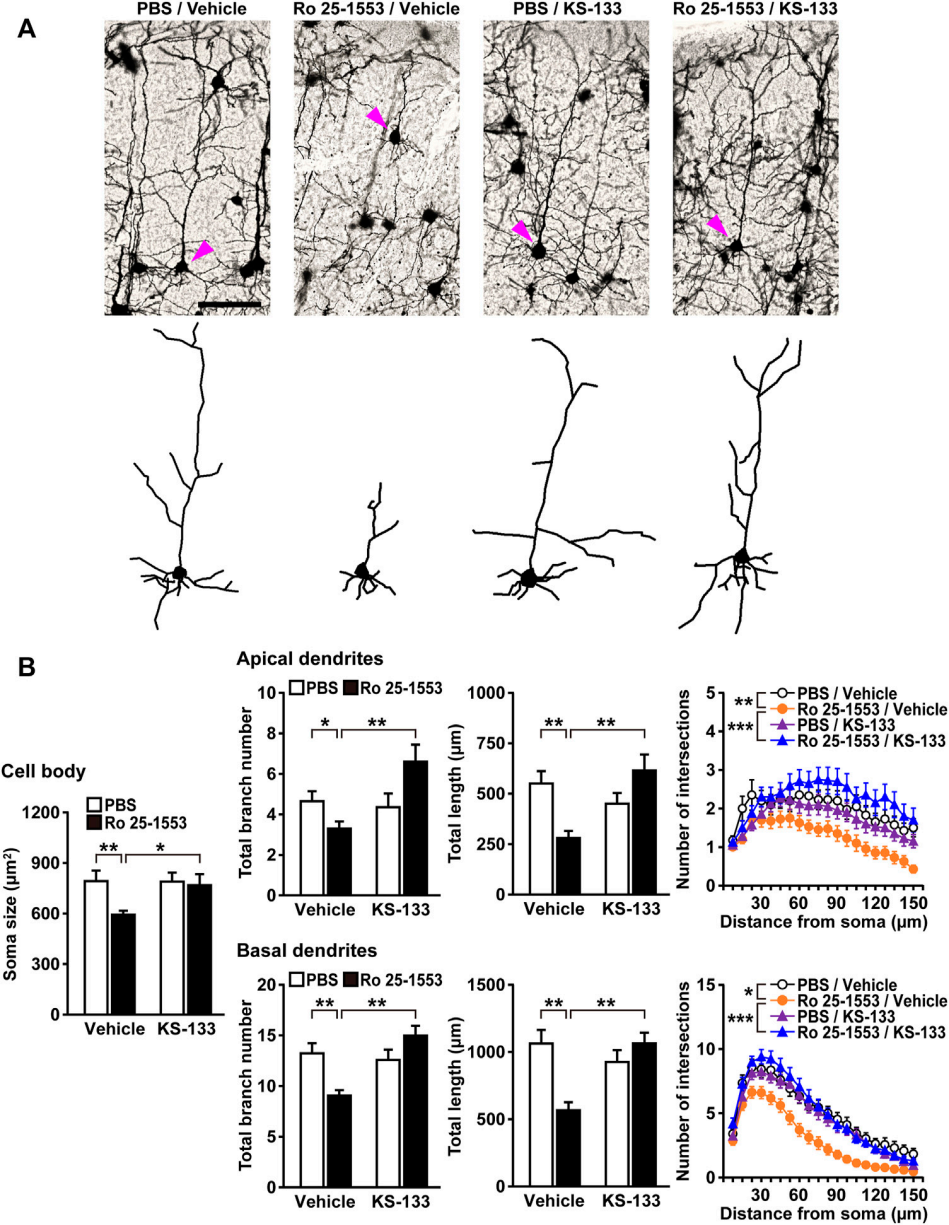
Alterations in the dendritic morphology of prefrontal pyramidal neurons in mice treated with Ro 25-1553 and KS-133 during the early postnatal period. Brain samples were obtained from mice after the novel object recognition test (Figure 5). **(A)** Golgi-stained pyramidal neurons and representative tracings of soma and dendrites. Scale = 100 μm. **(B)** The soma size (area), total branch number and length of apical and basal dendrites. The number of intersections of dendrites with 7.5-μm concentric spheres centered on the soma was measured by Sholl analysis. Results are expressed as the mean ± S.E.M. of 40 neurons from five mice per group. *p < 0.05, **p < 0.01, ***p < 0.001.

Because a reduced length of basilar dendrites and a reduced dendritic number have been found in layer 3 in prefrontal cortical areas [Brodmann area (BA) 10, BA 11, and BA 46] and the anterior cingulate cortex (BA 32) of schizophrenic patients (Glantz and Lewis, 2000; Kalus et al., 2000; Broadbelt et al., 2002; Black et al., 2004; Konopaske et al., 2014), we examined dendritic morphology in the prefrontal cortex of mice treated with Ro 25-1553 and KS-133 after the novel object recognition test (Figure 6). Figure 6A shows Golgi-stained pyramidal neurons and representative tracings of soma and dendrites in the prefrontal cortex. Ro 25-1553 reduced the cell soma size, total branch number and length of apical and basal dendrites of prefrontal cortex neurons (Figure 6B). These effects were counteracted by simultaneous administration of KS-133 with Ro 25-1553. Sholl analysis also revealed that the amount of dendritic material distal to the soma in apical (treatment × distance interaction: *F*_19, 1482_ = 1.957, *p* < 0.01) and basal (*F*_19, 1482_ = 1.750, *p* < 0.05) dendrites was decreased in Ro 25-1553-treated mice, which indicated a reduction in dendritic complexity. KS-133 prevented the morphological abnormalities in both apical (*F*_19, 1482_ = 3.261, *p* < 0.001) and basal (*F*_19, 1482_ = 3.877, *p* < 0.001) dendrites in the prefrontal cortex.

## Discussion

In this study, we optimized VIpep-3 to obtain derivatives and apply them *in vivo*. Using structural information of the extracellular domain of VIPR2, the C-terminal structure of VIP, and docking model of VIPR1/VIP, we constructed a molecular design concept. Additionally, by referencing the structure of VIP analogues such as Ro 25-1553, a bicyclization strategy was applied. The resulting KS-133 overcame most disadvantages of parental VIpep-3, such as vulnerability to protease degradation, and had high potency and selectivity for VIPR2. VIPR2 antagonist activity was improved in the Gq/calcium signaling pathway, even though the molecular weight was reduced from VIpep-3 (1941.1 g/mol) to KS-133 (1558.8 g/mol). Bicyclization would stabilize the peptide structure necessary for VIPR2 binding. For the Gs/cAMP signaling pathway, KS-133 showed a moderate antagonistic activity against VIPR2, whereas it did not exhibit the agonist activity. Importantly, KS-133 is active *in vivo*, inhibiting VIPR2-mediated CREB activation and preventing cognitive impairment in a pharmacological model of early postnatal VIPR2 overactivation, a relevant mouse model of schizophrenia (Ago et al., 2015; Ago et al., 2021). Notably, KS-133 had a different mechanism of action (MOA) from existing drugs that are effective mainly for positive symptoms by a common MOA that targets neurotransmitter receptors such as dopamine, serotonin, noradrenaline, and NMDA receptors. Because schizophrenia is a complex multifactorial disease, it is necessary to develop new therapeutic agents on the basis of its pathological mechanism.

Generally, drug discovery to target molecules in the CNS is harder than in peripheral tissues. Molecule exchange between the periphery and CNS is strictly restricted by the blood−brain barrier (BBB), especially molecules with a molecular weight (MW) of >400 g/mol. In addition to the MW limit, CNS drugs need to have high lipophilicity to cross the BBB (McMartin et al., 1987; Dong, 2018), but high lipophilic small molecules likely have undesirable side effects by potential off-target binding. Conversely, the MW of KS-133 was 1558 g/mol, which would avoid off-target side effects by VIPR2-selective binding but would make it difficult to cross the BBB. In the present study, phosphorylated CREB in the prefrontal cortex of neonatal mice at postnatal day (P) 12 was significantly increased by s.c. administration of VIPR2 agonist Ro 25-1553, which was suppressed by s.c. coadministration of KS-133. VIPR2 gene expression in the developing mouse brain displays a pronounced peak at P12 (Waschek et al., 1996) and VIPR2 is abundant in brain regions of cognitive circuitry, such as the prefrontal cortex (Vertongen et al., 1997; Joo et al., 2004; An et al., 2012). However, it was unclear whether the alterations in CREB phosphorylation were due to directly effects of s.c. injection of Ro 25-1553 and KS-133 on VIPR2 in the CNS. In another experiment, i.n. administration of BAY 55-9837, a VIPR2 agonist, also increased CREB phosphorylation in the prefrontal cortex of adult mice, which was suppressed by i.n. co-administration of KS-133. Intranasal administration is an attractive route for drug delivery to the brain because it allows direct transport of drugs from the nasal cavity to the brain parenchyma by bypassing systemic circulation (Thorne et al., 2004; Lochhead and Thorne, 2012; Iwasaki et al., 2019). It has been reported that about 1% of an administered molecule enters the CNS without crossing the BBB by administration to the nasal cavity, even those with a MW of 1000 g/mol (McMartin et al., 1987; Dong, 2018). There are some reports that the BBB becomes leaky in patients with psychiatric disorders such as schizophrenia and ASD (Patel and Frey, 2015; Najjar et al., 2017; Kealy et al., 2020). Additionally, some reports show that the PACAP/VIP family directly crosses the BBB (Banks et al., 2002; Dogrukol-Ak et al., 2004; Cabezas-Llobet et al., 2018; Cherait et al., 2021). In the separate experiment, the plasma and brain levels (*n* = 3, mean ± S.E.M.) of KS-133 at 80 min after intravenous administration of KS-133 (1 nmol/g) were 2.78 ± 0.08 μM and 0.035 ± 0.005 nmol/g tissue for adult male ICR mice, respectively, indicating that KS-133 can cross the BBB (unpublished data). KS-133 had a plasma elimination half-life of less than an hour. In the future, to clarify the mechanism and routes of how administered KS-133 targets the CNS, detailed pharmacokinetic studies of KS-133 in healthy mice and disease model mice using s.c., i.n., and intravenous administrations will be needed.

Cognitive deficits are considered a central feature of schizophrenia (Rund, 1998; Green et al., 2000; Bowie and Harvey, 2006). Additionally, clinical studies have shown a relationship between cortical thickness and cognitive performance in fronto–temporal brain regions in schizophrenia patients (Alkan et al., 2021) and several different whole brain voxel-based imaging techniques have identified the medial prefrontal cortex as a prominent site of abnormality in schizophrenia (Pomarol-Clotet et al., 2010). Thus, in the present study, we focused on recognition memory and dendritic morphology in the prefrontal cortex of early postnatally VIPR2-activated mice. We found that repeated administration of Ro 25-1553 during P1–14 caused cognitive impairment in adulthood and simultaneous treatment with KS-133 prevented this effect. In agreement with this observation, the same postnatally restricted Ro 25-1553 treatment reduced the total branch number and length of apical and basal dendrites of the prefrontal cortex neurons in mice. Additionally, Sholl analysis revealed reductions in dendritic complexity in both apical and basal dendrites. These *in vivo* morphological abnormalities were counteracted by concomitant administration of KS-133 with Ro 25-1553. Interestingly, Ro 25-1553-treated mice had smaller neuronal cell bodies than PBS-treated control mice, and this was also blocked by co-administration of KS-133. Postmortem studies have shown that pyramidal cell somal volume was reduced in layer 3 of the primary auditory cortex (BA 41) and auditory association cortex (BA 42) in schizophrenia (Sweet et al., 2003, 2004). Additionally, in layer 3 of dorsal prefrontal cortex, pyramidal cell somal volume, dendritic length, and spine density were diminished in concert in subjects with schizophrenia (Glantz and Lewis, 2000). Pyramidal cell somal volume has been suggested to be correlated with the extent of dendritic arborization and number of dendritic spines (Jacobs et al., 1997) and therefore might serve as a marker for alterations in additional components of cortical circuits. Taken together, these findings suggest that activation of VIPR2 during early postnatal development in mice leads to long-term impairment of cognition associated with changes in pyramidal cell size and dendritic morphology in the prefrontal cortex and that KS-133 has an *in vivo* VIPR2 antagonistic activity.

PACAP and VIP in peripheral tissues and the CNS play diverse roles in cell proliferation, differentiation, survival, maturation, and neuroprotection in both early development and adulthood through PAC1, VIPR1, and VIPR2 (Waschek, 2002; Falluel-Morel et al., 2007; Vaudry et al., 2009; White et al., 2010; Doan et al., 2011; Passemard et al., 2011; Solés-Tarrés et al., 2020). It is still not fully understood how the balance of timing and intensity of each receptor activation results in which phenotypes in individuals. Peptides acting on VIPR2 with selectivity, such as VIpep-3 (Sakamoto et al., 2018), Ro 25-1553 (Gourlet et al., 1997), Ro 25-1392 (Yung et al., 2003), and BAY 55-9837 (Tsutsumi et al., 2002), have been reported, but they are all agonists except for VIpep-3. Because KS-133 is a selective antagonist of VIPR2 *in vitro* and *in vivo*, it would be a good lead molecule for schizophrenia therapy and a tool compound to promote scientific research of VIPR2. The combination of a receptor-selective agonist/antagonist would be effective to investigate which time point of VIPR2 activation in the life cycle induces which phenotypes in the organism. Using VIPR2-knockout animals, it might be difficult to specify which expressed phenotypes are caused at which time point during the life cycle by which ligand (VIP and/or PACAP) and which site (peripheral and/or CNS). KS-133 would help to elucidate how differences in the therapeutic strategy to target VIPR2 from those of existing psychotropic drugs and whether VIPR2 inhibition exerts additive/synergistic effects to existing drugs.

Several clinical (Levinson et al., 2011; Vacic et al., 2011; Yuan et al., 2014; Li et al., 2016; Firouzabadi et al., 2017) and preclinical (Ago et al., 2015; Tian et al., 2019) studies have shown that high expression/overactivation of VIPR2 is linked to both schizophrenia and ASD. Additionally, Ro 25-1553 reduced axon and dendritic outgrowth in cultured cortical neurons by a protein kinase A (PKA)-dependent mechanism (Takeuchi et al., 2020). VIPR2 microduplication in mice elicited cognitive, sensorimotor gating, and social behavioral deficits preceded by an increase of striatal cAMP/PKA signaling and the disrupted early postnatal striatal development (Tian et al., 2019). Although the mechanisms by which overactive VIPR2 signaling may lead to psychiatric disorders are still not fully understood, activation of the cAMP/PKA pathway downstream of VIPR2 might be at least partly involved in neuronal development impairment and behavioral abnormalities. Therefore, in the case that personal genetic analysis reveals a CNV of VIPR2, administration of a VIPR2 inhibitor has the potential to be effective to both prevent the onset of the disease and possibly improve the pathological conditions of diseases when VIPR2 overactivity disrupts ongoing synaptic plasticity in the adult brain. At least, our study demonstrates that VIPR2 inhibition is effective against schizophrenia model mice established by VIPR2 overactivation.

In conclusion, we successfully generated KS-133 that may contribute to both the development of a novel drug candidate for the treatment of psychiatric disorders such as schizophrenia and acceleration of basic studies on VIPR2. Because current drug discoveries for the CNS are limited to small compounds, many drug targets of the CNS, such as class-B GPCRs, are considered intractable. Our study might be a valuable example demonstrating that such intractable targets can be druggable targets using a highly molecular designed peptide.

## Data Availability

The raw data supporting the conclusions of this article will be made available by the authors, without undue reservation.

## Glossary

Ac: acetyl group
Arg: l-arginine
ASD: autism spectrum disorder
Asp: l-aspartic acid
BBB: blood−brain barrier
cAMP: cyclic adenosine monophosphate
CNV: copy number variation
CREB: cAMP-response element-binding protein
CNS: central nervous system
Cys: l-cysteine
_c_(XZ): cyclization between amino acids X and Z
_c_[XZ]: cyclization between amino acids X and Z
Dap: l-2,3-diaminopropionic acid
DCM: dichloromethane
GPCR: G protein-coupled receptor
Ile: l-isoleucine
Leu: l-leucine
Lys: l-lysine
MALDI-TOF: MS, matrix-assisted laser desorption ionization-time of flight mass spectrometry.
MOA: mechanism of action
MW: molecular weight
NMDA: *N*-methyl-d-aspartate
PAC1: pituitary adenylate cyclase-activating polypeptide type-1 receptor
PACAP: pituitary adenylate cyclase-activating polypeptide
PKA: protein kinase A
Pro: l-proline
RP-HPLC: reverse phase-high performance liquid chromatography
TFA: trifluoroacetic acid
Thr: l-threonine
Tyr: l-tyrosine
VIP: vasoactive intestinal peptide
VIPR: vasoactive intestinal peptide receptor
