# Supplementary material for: Generation of KS-133 as a Novel Bicyclic Peptide with a Potent and Selective VIPR2 Antagonist Activity that Counteracts Cognitive Decline in a Mouse Model of Psychiatric Disorders
Source: Front Pharmacol. 2021 Nov 4;12:751587. doi: 10.3389/fphar.2021.751587 (PMC8607231; doi:10.3389/fphar.2021.751587)
Supplement: Supplementary file 1 [file Table1.DOCX]

**Supplementary Table 1.** Analytical data of peptides in this study.

RP-HPLC Mass (Linear mode) (g/mol)

Name T_ret_ (min) Purity (%) Calc. Obsv.^Voltage polarity^

VIpep-3 ^A^12.674 98.11 1941.4 1941.954^POS^

KS-132 ^A^13.051 100.00 1505.8 1505.422^POS^

KS-133(monocyclic) ^A^15.125 100.00 1576.9 1576.047^POS^

KS-133 ^B^10.617 100.00 1558.8 1558.705^NEG^

Retention times and purities of peptides were characterized by RP-HPLC using a SunFire C18 5 µm column (4.6 × 150 mm) at a wavelength of 220 nm under four linear gradient conditions: (A) 10%−60% acetonitrile or (B) 20%−90% acetonitrile in water with 0.1% TFA for 20 min (1 mL/min). Molecular weights of peptides were determined by an autoflex speed MALDI-TOF mass spectrometer.
